# Establishment and Analysis of a Combined Diagnostic Model of Alzheimer's Disease With Random Forest and Artificial Neural Network

**DOI:** 10.3389/fnagi.2022.921906

**Published:** 2022-06-30

**Authors:** Dazhong Sun, Haojun Peng, Zhibing Wu

**Affiliations:** The First Clinical Medical School, Guangzhou University of Chinese Medicine, Guangzhou, China

**Keywords:** Alzheimer's disease, random forest, artificial neural network, GEO, diagnostic model

## Abstract

Alzheimer's disease (AD) is a neurodegenerative condition that causes cognitive decline over time. Because existing diagnostic approaches for AD are limited, improving upon previously established diagnostic models based on genetic biomarkers is necessary. Firstly, four AD gene expression datasets were collected from the Gene Expression Omnibus (GEO) database. Two datasets were used to establish diagnostic models, and the other two datasets were used to verify the model effect. We merged GSE5281 with GSE44771 as the training dataset and found 120 DEGs. Then, we used random forest (RF) to screen 6 key genes (KLF15, MAFF, ITPKB, SST, DDIT4, and NRXN3) as being critical for separating AD and normal samples. The weights of these key genes were measured, and a diagnostic model was created using an artificial neural network (ANN). The area under the curve (AUC) of the model is 0.953, while the accuracy is 0.914. In the final step, two validation datasets were utilized to assess AUC performance. In GSE109887, our model had an AUC of 0.854, and in GSE132903, it had an AUC of 0.810. To summarize, we successfully identified key gene biomarkers and developed a new AD diagnostic model.

## Introduction

Alzheimer's disease (AD) is a type of chronic degenerative brain illness marked by central nervous system disorder that primarily affects people in their forties and fifties (Scheltens et al., 2021). The main clinical feature of AD is memory impairment, which may be accompanied by aphasia and personality behavior changes (Scheltens et al., 2016). Pathophysiological changes in AD may begin years before any clinical symptoms appear and may progress all the way to severe cognitive impairment (Aisen et al., 2017). As a result, AD cannot be identified just on the basis of clinical characteristics, and researchers have made exhaustive efforts to identify AD using clinical and biomarker data (Delaby et al., 2022). Understanding of AD has grown significantly over the past few decades while also highlighting the disease's complexity (Chen, 2018). Imaging technologies, cognitive level identification, and various fluid biomarkers are now used to diagnose AD (Reitz, 2015; Blennow and Zetterberg, 2018; Sun et al., 2018). It is becoming more apparent that AD is a disease with a complex regulatory network that is becoming increasingly complex (Veitch et al., 2019). As a result, more precise diagnostic and treatment targets for AD are urgently needed. The rapid advancement of microarray and high-throughput sequencing technologies in the last decade has suggested a reliable and widespread method for decoding inherited and epigenetic determinants of disease. At the same time, it also provides a lot of evidence for the diagnosis and treatment of various diseases (Kulasingam and Diamandis, 2008). Although genetic risk markers have been identified that can be used to predict and diagnose AD, their power may be limited because of the complexity of the genetic structure (Zhu et al., 2020). In diagnostic models, the use of multiple biomarkers has been shown to improve success rates significantly (Vilhjálmsson et al., 2015). In recent years, the primary difficulty in constructing a classification model based on gene expression data has been choosing the most significant index or feature for classification. This problem can be solved using a variety of machine learning techniques (Kursa, 2014; Tian et al., 2020; Xie et al., 2020). These algorithms have made significant contributions to the classification of gene expression data, disease detection, cell migration, and microbiome research when used alone or in combination (Hsieh et al., 2011; Kong and Yu, 2018; Zhang et al., 2018; Janßen et al., 2019).

Using the key genes screened from datasets in the GEO database, we created an AD diagnosis model. It was first determined which genes were most important for AD classification using RF. A genetic diagnostic model for AD was then built using these key genes by artificial neural networks. We evaluated the performance of the diagnosis model with independent validation datasets to confirm its accuracy and performance.

## Materials and Methods

### Study Design

**Figure 1:**
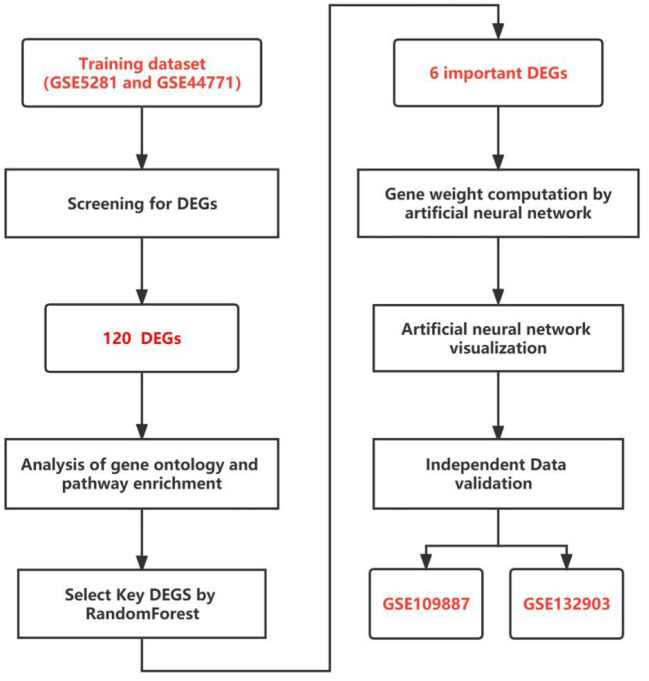
The flow chart of the study.

For the differentially expressed genes (DEGs) screening, the GSE5281 dataset was merged with the GSE44771 dataset as the training dataset (step 1). We went on to analyse gene ontology and pathway enrichment (step 2). Then, we screened the key genes using RF classification (step 3). Following the computation of gene weights (step 4), an ANN model was developed (step 5). In the end, GSE109887 and GSE132903 datasets were used to conduct further validation (step 6). All statistics are computed by R software version 4.1.3. Figure 1 depicts the entire research flow.

### Data Selection and Processing

Datasets in this study were obtained from the GEO database, which stores information about how genes are expressed using high-throughput methods. It was created by the National Center for Biotechnology Information (NCBI) (https://www.ncbi.nlm.nih.gov/geo/query/acc.cgi). The keywords “AD, normal” or “AD, health” were used in this study to conduct a broad search through the NCBI database platform. The type of datasets we chose was expression profiling by array, and the type of organisms was homo sapiens. The sample size of the dataset is greater than 60. We used the ComBat function in R package sva (Varma, 2020) to remove the batch effect of data from different platforms. The log2-transformed quantile-normalized signal intensity of these datasets was rectified, and the corrected results were outputted.

### Screening for DEGs

Using traditional Bayesian data analysis, the R package limma (Ritchie et al., 2015) was utilized to screen DEGs of the training dataset. Adjusted *P* values less than 0.05 and logFoldChang (logFC) greater than 1 were established as the significance criteria for DEGs. The DEGs heatmap was created using the R package pheatmap. The volcano plot was created using the R package ggplot2 (Ito and Murphy, 2013).

### Analysis of Gene Ontology and Pathway Enrichment

Gene ontology and pathway analysis are utilized for the purpose of interpreting gene expression data. An online comprehensive gene set enrichment web tool, EnrichR (https://maayanlab.cloud/EnrichR), was used in our study to conduct gene ontology and pathway enrichment analyses. Gene ontology, including biological processes, cellular components, and molecular functions, was analyzed using EnrichR. In addition, we used KEGG pathway 2021, WikiPathways 2021, and Retcome 2016 as classification sources for pathways to identify gene common pathways. EnrichR used the logarithm of the *P*-value and the z-score to create a combined score. We ranked them in order of the combined score and showed them in bar charts.

### Random Forest Screening for Key Genes

We screened the key genes using random forest by R package random Forest (R project, 2022). In order to determine the lowest error rate and best stability tree number as the optimal parameter, each error rate for 1–200 trees was calculated. After that, a random forest was used to screen key genes, and the Gini coefficient method was used to calculate the dimensional significance value. The AD key genes for ANN model development were selected from the top 30 DEGs with a significance value greater than 6. The key genes in the training dataset were put into new groups based on their unsupervised hierarchical clusters, and the heatmap was generated using the R package pheatmap (Hu, 2021).

### Artificial Neural Network for Building an AD Classification Model

First, the DEG expression data was converted to a Gene Score table based on the expression level. A comparison was made between the median of all sample expression values and the expression value of a single gene in a given sample. If the expression value of the up-regulated gene is greater than 0, it will be given a 1; otherwise, it will be given a 0. Likewise, if a down-regulated gene's expression value is higher, it will be given a value of 0; otherwise, it will be given a value of 1. AD was the outcome variable, and cases were assigned a 1 while controls were assigned a 0. The R package neuralnet (Beck, 2018) was used to create an ANN model based on the Gene Score table we constructed. The model parameter was set to 5 hidden layers. R package Caret (Nachid and Boussiala, 2021) was used to calculate 5-fold cross-validation of the ANN model in order to optimize the model and reduce overfitting. The confusion matrix function calculated the accuracy of the results. Using the R package pROC (Robin et al., 2011), we calculated the areas under the receiver operating characteristic curve (AUC).

### Verification Using Validation Datasets

On two separate validation datasets (GSE109887 and GSE132903), the ANN model was tested for effectiveness verification. The AUC was calculated using the R package pROC.

## Results

### Identification of DEGs

**Figure 2:**
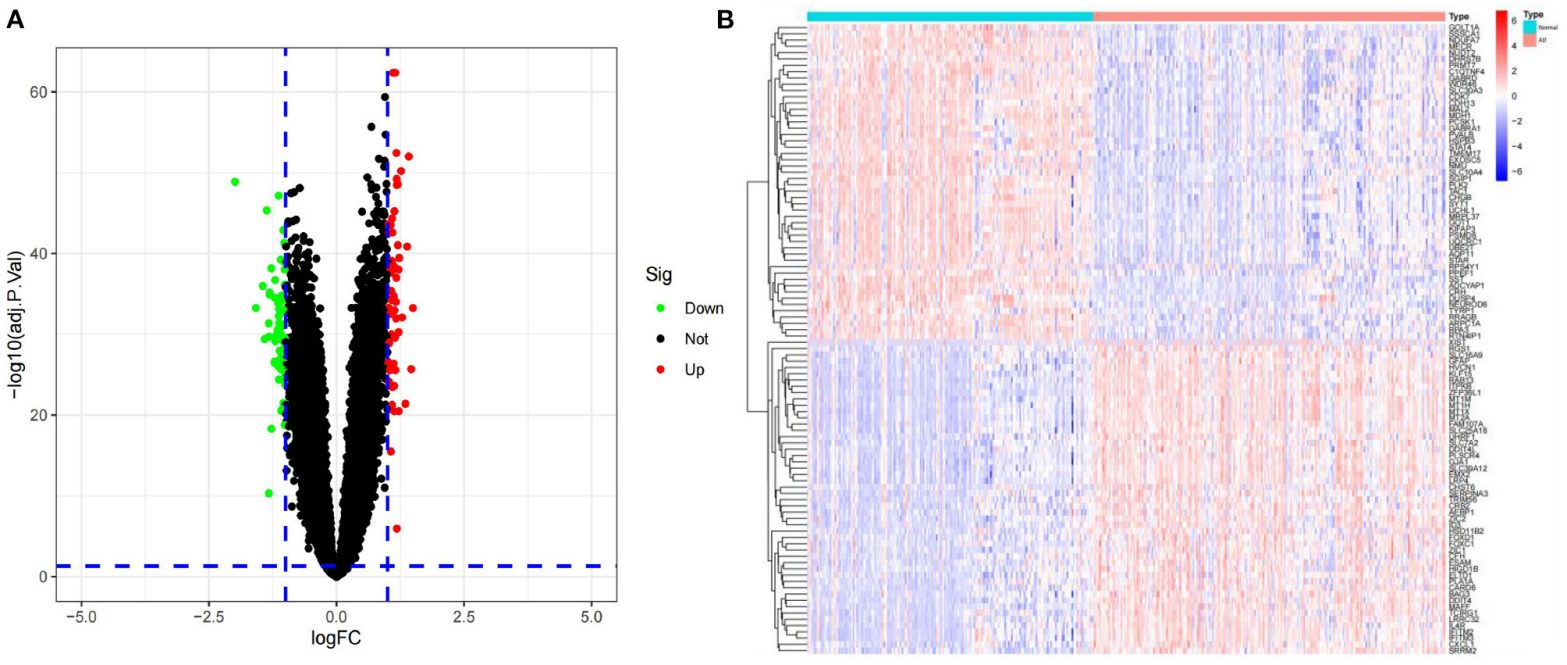
**(A)** Volcano plots of all DEGs in the training dataset. Green spots represent down-regulated genes, while red spots represent up-regulated genes. **(B)** All of the DEGs are represented as a heatmap. Up- and down-regulated genes are marked on the map. Red samples indicate AD, while blue samples indicate normal. Red blocks indicate high-expressed genes, and blue blocks low-expressed genes.

GSE5281 was a dataset including 74 AD samples and 87 control samples. Brain samples were collected from three Alzheimer's Disease Centers. Gene expression was analyzed using Affymetrix U133 Plus 2.0. GSE44771 was a dataset including 101 AD samples and 129 control samples. Brain samples were collected through the Harvard Brain Tissue Resource Center. Gene expression was analyzed using Rosetta/Merck Human 44k 1.1 microarray. GSE109887 was a dataset including 32 AD samples and 46 control samples. Brain and blood samples were collected through University Medical Center Göttingen. Gene expression was analyzed using Illumina HumanHT-12 v4 BeadChip. GSE132903 was a dataset including 98 AD samples and 97 control samples. Brain samples were collected through America Translational Genomic Research Institute. Gene expression was analyzed using Illumina Human HT-12 v4 arrays. Details about four datasets are shown in Table 1. Two datasets (GSE5281 and GSE44771) were combined to create a training dataset with a large sample size. Meanwhile, GSE109887 and GSE132903 were set as validation datasets. The training dataset was screened and eventually identified 120 significant DEGs related to AD based on logFC>1 and adjusted *P-*value < 0.05. A volcano map was used to depict the expression status of all DEGs in the training dataset (Figure 2A). The difference between upregulated and downregulated genes were distinct. Using the heatmap, we can see which of the DEGs have the most upregulated gene expression compared to the control group (Figure 2B).

**Table 1:** The information of training/validation datasets.

**Dataset ID**	**Platform**	**AD**	**Normal**	**Total**	**Group**
GSE5281	GPL570	74	87	161	Training
GSE44771	GPL4372	101	129	230	Training
GSE109887	GPL10904	32	46	78	Validation
GSE132903	GPL10558	98	97	255	Validation

### Analysis of Gene Ontology and Pathway Enrichment

**Figure 3:**
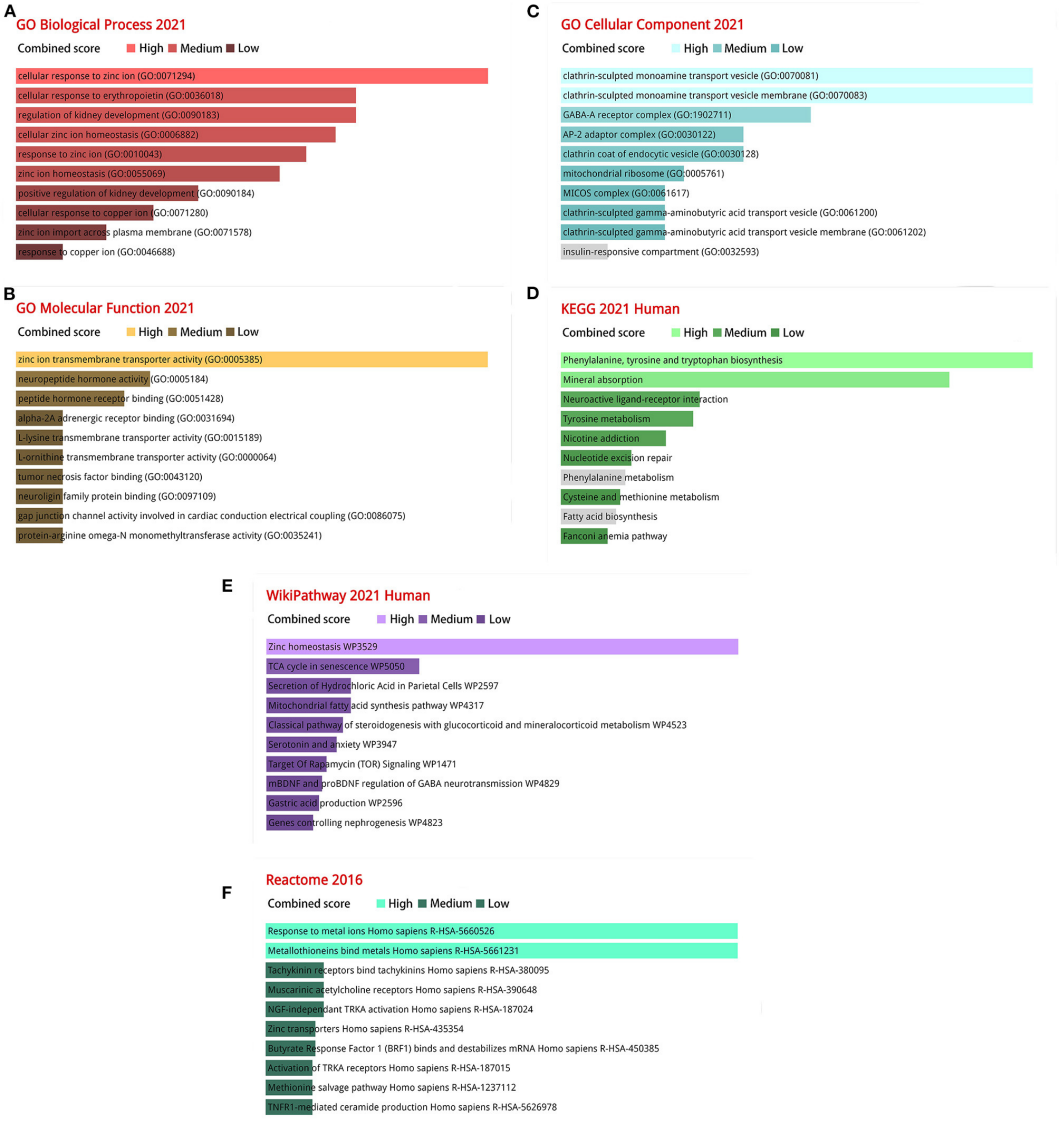
The bar charts of ontological and pathway enrichment analysis of DEGs. **(A)** Go biological processes; **(B)** Go molecular function; **(C)** Go cellular component; **(D)** KEGG human pathway 2021; **(E)** Wikipathway 2021; **(F)** Reactome pathway 2016.

We analyzed the ontology and pathway enrichment for the 120 DEGs. For the Biological Process subsection, the results demonstrate that the DEGs were significantly enhanced in cellular response to zinc ion. Molecular function subsection data indicated a zinc ion transmembrane transporter activity involved in the DEGs. The Cellular Component analysis revealed that clathrin-sculpted monoamine transport vesicle played a significant role. It showed the Phenylalanine, tyrosine and tryptophan biosynthesis, Zinc homeostasis and Response to metal ions interaction with the most important genes according to the KEGG, WikiPathway and Reactome pathway. The combined scores rank for GO terms and analysis results from various pathway databases are shown in Figure 3.

### Random Forest Screening for Key Genes

**Figure 4:**
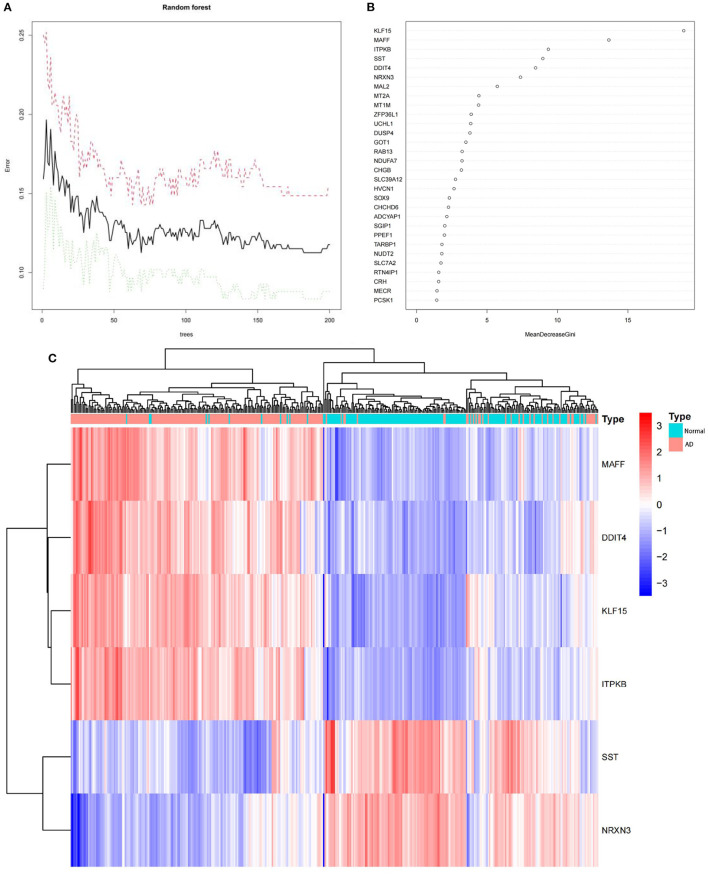
**(A)** The correlation plot between the number of RF trees and model error. The error rate is stable when the number of RF trees is around 190. **(B)** The Gini coefficient method in a random forest classifier yielded the following results. The importance index is on the x-axis, and the genetic variable is on the y-axis. **(C)** The heatmap of six key genes generated by random forest. The red band indicate AD, while the blue band indicate normal. Red blocks indicate high-expressed genes, and blue blocks low-expressed genes.

To obtain key genes, we fed the 120 DEGs listed above into the RF classifier. Based on the correlation plot between the number of RF trees and model error (Figure 4A), we chose 190 trees as the final model's parameter. We then identified six genes with a significance >6 as candidate genes for further analysis. According to Figure 4B, KLF15 was the most significant variable, followed by MAFF, ITPKB, SST, DDIT4, and NRXN3. Figure 4C show that in 120 DEGs from the training dataset, the six genes were able to identify AD samples. MAFF, DDIT4, KLF15, and ITPKB genes were a group of genes whose expression was low in normal samples and high in AD samples. On the other hand, SST and NRXN3 belonged to a different cluster. In normal samples, they were expressed at high levels, but in AD samples, they were expressed at low levels.

### Construction of the ANN Model

**Figure 5:**
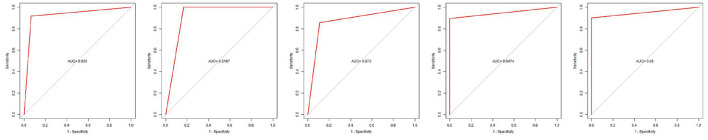
Five-fold cross-validation verifies ROC curve results.

**Figure 6:**
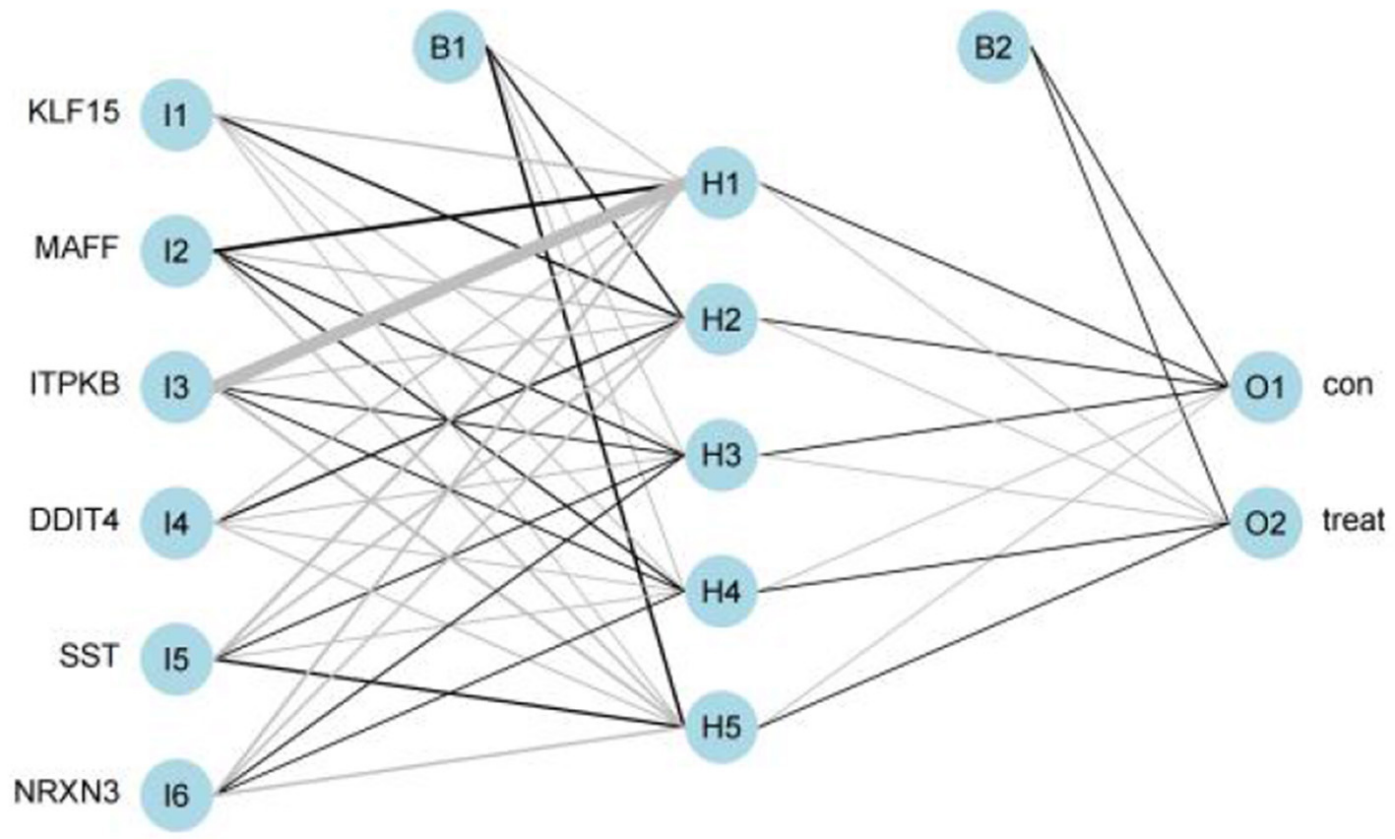
Visualization of artificial neural networks' results.

**Figure 7:**
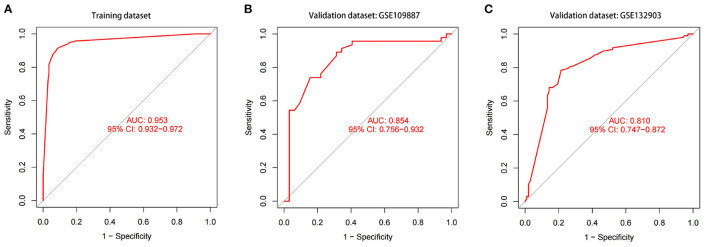
The ROC curves and their respective AUC values are utilized to evaluate the performance of the ANN model in training and validation datasets. **(A)** GSE5281 and GSE44771 datasets. **(B)** GSE109887 dataset. **(C)** GSE132903 dataset.

We got a Gene Score table with 6 lines of samples, 391 columns, and a column for the AD outcome variable (case/control). We built an ANN model based on the Gene Score table. Six input layers, five hidden layers, and two output layers were set for the ANN. Each result of the 5-fold cross-validation is presented by ROC curves (Figure 5), while the accuracy is shown in Table 2. The model's reliability was demonstrated by the fact that the average AUC of the 5-fold cross-validation results exceeded 0.90. Finally, we built an ANN model for classifying gene expression data between AD and control samples based on the information presented above (Figure 6). The overall AUC of this model is 0.953, and its accuracy is 0.914 (Figure 7A).

**Table 2:** Five-fold cross-validation results.

	**Accuracy**	**AUC**
Cross validation 1	0.9231	0.925
Cross validation 2	0.9231	0.9167
Cross validation 3	0.8718	0.873
Cross validation 4	0.95	0.9474
Cross validation 5	0.9487	0.95

### Validation of the ANN Model

The model's prediction accuracy was 0.854 in GSE109887 and 0.810 in GSE132903, indicating that the ANN is stable in diagnosing AD (Figure 7). These findings demonstrate that we successfully developed an AD diagnostic model based on the differential gene expression of AD and normal samples.

## Discussion

Over the last century, advances in AD research have led to the development of increasingly effective treatments (Sun et al., 2018). However, the specific mechanisms of AD development remain unknown. It is almost impossible to make an early clinical diagnosis of AD because the symptoms overlap with those of other neuropathological diseases. Identifying critical diagnostic and prognostic biomarkers for AD remains critical. Advancements in machine learning and public gene expression data make it feasible to infer biomarkers for disease diagnosis and prognosis (Ramakrishnan et al., 2019).

In our study, we combined an AD diagnostic model with random forest and an artificial neural network that could distinguish AD samples from normal samples. Diagnostic evidence for diseases like AD is being bolstered by advances in high-speed bioinformatics. To identify DEGs of AD, we first combined two GEO datasets (GSE5281 and GSE44771). Then analyzed the gene ontology and pathway enrichment. According to the GO and pathway enrichment analysis, the DEGs are related to a vast array of GO terms and pathways, reflecting the pathogenesis' dynamics and complexity. There are already many studies supporting our findings. Prior research has suggested a connection between zinc ion and the occurrence of AD. The new research has uncovered a list of essential zinc ion transmembrane transporters whose mRNA or protein levels were found to be abnormally altered at various stages of AD (Xu et al., 2019). Changing zinc levels, especially at the synapses, have been suggested as a possible cause of cognitive changes that come with aging and AD (Hancock et al., 2014). Aged brains have been predicted to have less efficient homeostasis mechanisms and molecules for zinc ions (Bertoni-Freddari et al., 2006). The best way to understand an organism's internal changes is to conduct a pathway analysis. The disruption of phenylalanine metabolism in the hippocampus could be an important factor in the progression of AD (Liu et al., 2021). In AD, the peripheral modulation of tyrosine phosphorylation signaling could be investigated as a potential diagnostic marker (Mallozzi et al., 2020). It is possible that the pathogenesis of AD is influenced by immune activation-induced tryptophan degradation (Widner et al., 2000). Dyshomeostasis of zinc in the brain contributes to AD. Excess zinc is toxic to neuronal cells (Li and Wang, 2016). Homeostasis of metal ion levels is essential for normal physiological processes. Researchers have discovered a link between AD and an imbalance in the metal ions in the brain (Wang L. et al., 2020).

Further performance of RF classification screened out 6 key genes, namely, KLF15, MAFF, ITPKB, SST, DDIT4, and NRXN3. Previous research has supported our findings. Kruppel Like Factor 15 (KLF15) is a member of the Sp/KLF family of zinc-finger transcription factors. This family has been linked to controlling many cellular processes, such as cell growth, differentiation, normal development, and even cancer. It inhibits the growth of neurons (Otteson et al., 2004; Wang X. et al., 2020). MAF BZIP Transcription Factor F (MAFF) is upregulated in all tissues in AD. It can potentiate antioxidation inhibition and may be a potential therapeutic target in AD (Wang et al., 2017; Wang X. et al., 2020). Inositol (1,4,5) trisphosphate 3-kinase B (ITPKB) is an essential regulator in AD that plays a role in the apoptosis of neuronal cells, the processing of APP and the phosphorylation of tau (Stygelbout et al., 2014). Somatostatin (SST) receptor levels are lower in AD. SST-releasing neurons are often found near plaques. Its' expression levels decline with age (Beal et al., 1985; Roberts et al., 1985; Saito et al., 2005; Koivisto et al., 2007; Xue et al., 2009; Lau et al., 2017). Upregulation of DNA damage-inducible transcript 4 (DDIT4), a stress-regulated protein, can cause neuronal trigger death. It has been identified as a biomarker for AD (Pérez-Sisqués et al., 2021). Neurexin 3 (NRXN3) is a type of presynaptic adhesion molecule that regulates neurotransmitter release and specifies neuron synapses. In AD patients, NRXN3 expression is reduced. Dysregulation of presynaptic NRXN3 expression and splicing may promote neuron inflammation in the AD brain (Hishimoto et al., 2019). These studies demonstrated that the 6 key genes could be used as key biomarkers of AD.

The highlight of our study is the innovative combination of RF and ANN methods which yielded excellent results in terms of predictive power. Several other diseases, including ulcerative colitis, heart failure, and polycystic ovary syndrome, have already benefited from this innovative research technique (Li et al., 2020; Tian et al., 2020; Xie et al., 2020). Prior to this, a few AD prediction models based on methylated gene biomarkers had been developed (Ren et al., 2020; Mahendran and PM, 2022). However, some problems exist in these studies, such as small sample size or general prediction effect of the established models. Our model performed better on the validation datasets GSE109887 and GSE132903, with AUC of 0.854 and 0.810, indicating it is more suitable for AD classification.

Even so, there are some limitations in our research. Although we used two datasets with more samples to build a model, it is still not a big data sample for machine learning, and we can include more research data in the training set in the future. Overfitting in machine learning is objective and cannot be eliminated, even if we use 5-fold cross-validation in the modeling process to minimize overfitting. Checking for overfitting is helpful, but it does not solve the problem. This means that although we get a good model effect on the validation set, the actual generalization ability may not be good due to the appearance of noise in reality. So this still means that we need to include more research data to test the reliability of the model in the future.

## Conclusions

To summarize, our thorough examination of AD datasets from GEO revealed KLF15, MAFF, ITPKB, SST, DDIT4, and NRXN3 as potential diagnostic biomarkers. Based on machine learning algorithms employing RF and ANN, a diagnostic model for AD was created that demonstrated excellent prediction performance.

## Data Availability Statement

The datasets presented in this study can be found in online repositories. The names of the repository and accession numbers can be found below: https://www.ncbi.nlm.nih.gov/geo/ GEO accession numbers: GSE5281, GSE44771, GSE109887 and GSE132903.

## Author Contributions

DS: data collation and drafting the manuscript. HP: technical review and revision of data analysis. ZW: project administration and funding support. All authors have read and agreed to the published version of the manuscript.

## Funding

This research was funded by the National Key R&D Program of China and Ministry of Science and Technology of China, Grant Number 2018YFC2002504.

## Conflict of Interest

The authors declare that the research was conducted in the absence of any commercial or financial relationships that could be construed as a potential conflict of interest.

## Publisher's Note

All claims expressed in this article are solely those of the authors and do not necessarily represent those of their affiliated organizations, or those of the publisher, the editors and the reviewers. Any product that may be evaluated in this article, or claim that may be made by its manufacturer, is not guaranteed or endorsed by the publisher.
